# Review on Disasters and Lower Limb Venous Disease

**DOI:** 10.3400/avd.ra.21-00026

**Published:** 2021-12-25

**Authors:** Sergio Gianesini, Erica Menegatti, Oscar Bottini, Yung-Wei Chi

**Affiliations:** 1 Department of Translational Medicine, University of Ferrara, Ferrara, Italy; 2 Department of Surgery, Uniformed Services University of Health Sciences, Bethesda, MD, USA; 3 Phlebology and Lymphology Service, German Hospital of Buenos Aires, Buenos Aires, Argentina; 4 Department of Internal Medicine, University of California Davis, CA, USA

**Keywords:** disasters, earthquake, burns, intoxication, venous disease

## Abstract

As per the World Health Organization, a *disaster* is defined as “an event that occurs in most cases suddenly and unexpectedly, causing severe disturbances to people or objects affected by it, resulting in the loss of life and harm to the health of the population.” A number of health issues are often reported following disasters, such as physical and psychological trauma, infections, malnutrition, and cardiovascular events. Among these, venous thromboembolism is deemed serious and thus should be taken into consideration. Indeed, its risk has been demonstrated to increase following earthquakes, floods, burns, and intoxications. The recent coronavirus pandemic summarizes some of the main triggering factors involved in acute and chronic venous disease development in a disaster setting: inflammation, infection, lockdown-induced reduced mobility, potential malnutrition, and overweight.

Proper venous risk assessment and guideline application have been determined to be essential in disaster management, particularly in the current time in which sheltering could lead to a potential exacerbation of the pandemic, which can only increase the risk for venous thrombotic diseases.

Global scientific teamwork is needed to make the recommendations as evidence-based and as homogeneous as possible among continents.

In this present review, we focus on how earthquakes impact venous thromboembolism, including an analysis of other disaster-related conditions, such as burns and intoxication. (This is a review article based on the informative seminar of the 40th Annual Meeting of Japanese Society of Phlebology.)

## Introduction

According to the World Health Organization, a disaster is defined as a “sudden phenomenon of sufficient magnitude to overwhelm the resources of a hospital, region, or location requiring external support.”^1)^

Disasters are defined as “natural” whenever related to geophysical, meteorological, hydrological, climatological, or biological phenomena. On the contrary, disasters can be “man-made” if related to criminal terrorist attacks or technological incidents.

The latter may be referred in an industrial or transportation context.^2)^ The different types of disaster can be interconnected, for example, the 2011 Tohoku earthquake that led to a tsunami, which consequently led to the Fukushima Daiichi nuclear disaster.

Disasters are far more frequent than usually thought. In fact, more than 20,000 mass disasters have occurred since 1900, which resulted in 1.3 million deaths and 4.4 billion individuals in need of emergent assistance.

Floods, storms, droughts, heatwaves, and other extreme weather events have been accounted for 91% of these disasters.^3)^

The disasters may impact global health at multiple levels: direct trauma, challenging weather conditions, and direct contact with animals and insects in precarious sheltering leading to possible outbreaks of contagious infections. Foodborne illnesses, malnutrition, and psychological casualties should also to be taken into consideration. Lower limb chronic venous disease is an extremely frequent pathology, affecting more than half of the adult population in various severities.^4)^

This condition leads to a significantly increased risk of venous thrombosis.^5,6)^ An increased incidence of venous thromboembolism in subjects exposed to disasters, in particular of geophysical nature (earthquakes and tsunami), has been reported for decades now.^7)^

Thus, in this present review, we aim to analyze the available literature on the topic of how disasters impact venous disease, focusing on earthquakes, while also examining the roles of burns and intoxications.

## Materials and Methods

This present review methodology was done in accordance to the Quality of Reporting of Meta-analyses (QUOROM) indications^8)^ together with the PRISMA (Preferred Reporting Items for Systematic Reviews and Meta-Analyses) statement^9)^ and its most recent update.^10)^

### Searching

The literature search was performed in PubMed, Embase, Cinahl, and the Cochrane Library up to May 30, 2020. It focused on all papers dealing with disaster conditions and venous disease by using the following mesh terms: “disasters,” “venous thrombosis,” “varicose veins,” “embolism,” “embolism and thrombosis,” and “pulmonary embolism.” The papers were screened on their abstracts and, if considered suitable, entirely reviewed for inclusion. “Related articles” links and references of the assessed papers were evaluated as well.

### Selection

The review focused on topics dealing with venous disease (thromboembolism and/or chronic venous disease) and earthquakes or tsunami or flood. Further narrative search was performed on the topic on how burns and intoxication impact lower limb venous disease. No specific restrictions were made on the population type, number of enrolled subjects, follow-up length, and publication year. Only papers written in English were included.

Contributions coming from not indexed and/or from not English written journals were included only if considered of significant value for the revision.

Venous thromboembolism incidence and risk were considered as the main outcome.

### Validity assessment

Maximum validity was given to randomized trials including concealed allocation, assessors blinding, and longer than 1-week follow-up.

### Data abstraction

A reviewer (SG) independently screened the titles and abstracts for eligibility. The selected full papers are then assessed by three independent authors (SG, YWC, and EM).

Eventual disagreement between the reviewers was solved by a fourth reviewer (OB).

Data extraction was performed in duplicate by both reviewers using a standardized form, reporting the study description, type, and main results.

### Study characteristics

The review included randomized controlled trials, non-randomized controlled trials, cohort and case-control studies, retrospective investigations, and expert opinion articles.

## Results

**Figure 1:**
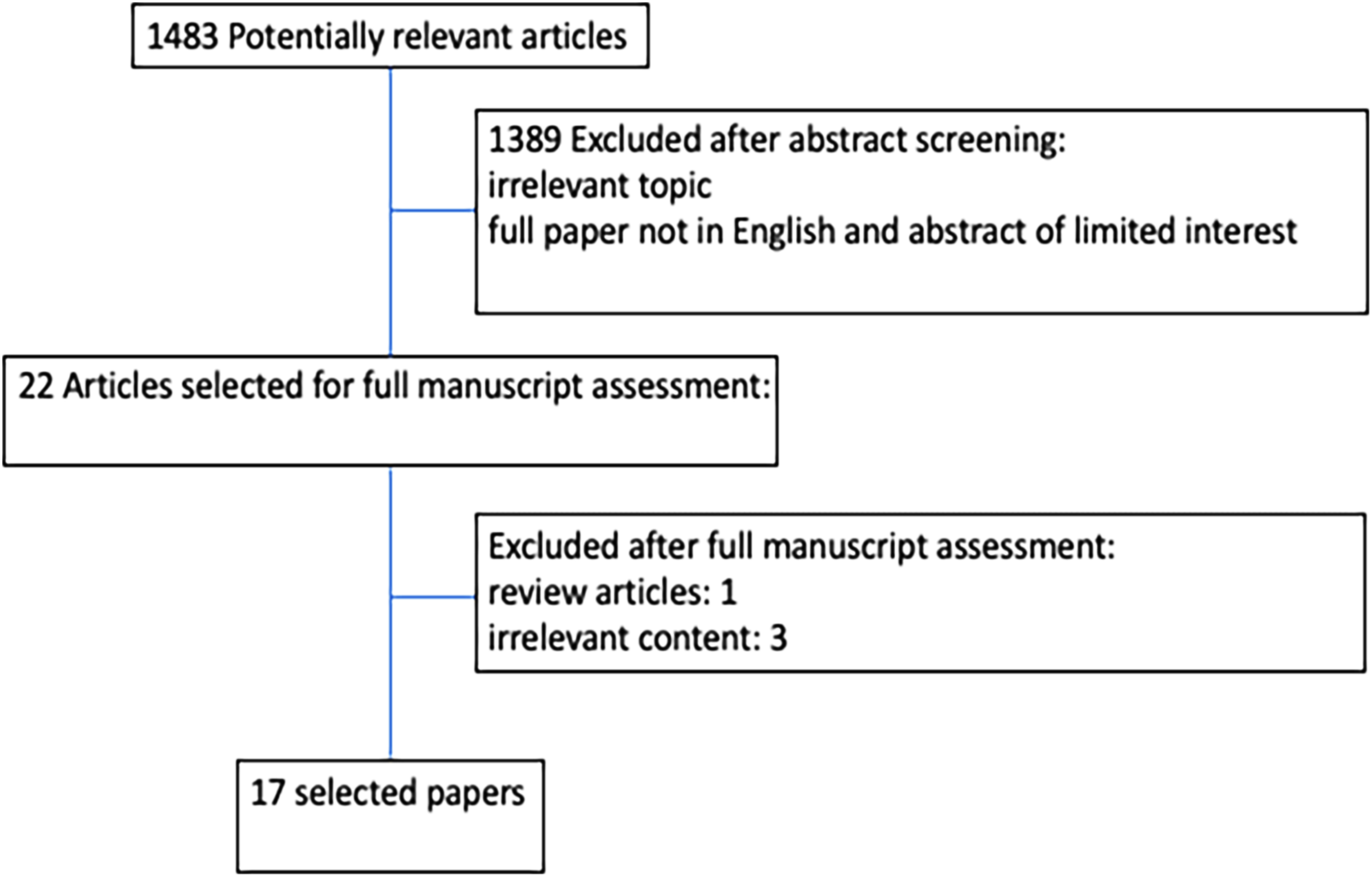
Fig. 1 Systematic selection of publications related to earthquakes and venous thromboembolism.

**Figure 1** reports the systematic selection process for publications related to earthquakes and venous thromboembolism. From an initial pool of 1483 references, 22 represented articles were deemed worthy to be assessed in their entireness. After a detailed revision, the final selection included 17 publications.

**Table 1** shows the main features of the selected investigations. These publications focused on deep venous thromboembolism incidence following earthquakes. Among these papers, 11 also included analysis on the possible risk factors associated with thromboembolism following the disaster.^11–21)^

**Table 1:** Table 1 Selected publications dealing with earthquake impact on venous disease

First author, year	Disaster	Population	DVT	PE	Assessment	F-up	Identified risk factors	Study design
Sato K, 2019	2016 Kumamoto Earthquake	1663	10.3%	NR	Questionnaire Portable US D-dimer	1 m	Age ≥70 y.o. Sleep medication Edema Varicose veins	Consecutive case series
Watanabe H, 2008	2004 Niigata earthquake	NR	NR	9 times increase	CT	1 m	No statistical calculation performed. Suggested role of: Automobile sheltering	Consecutive case series
Inoue K, 2006	2004 Chuetsu District earthquake	NR	NR	9 cases	CT	NR	No statistical calculation performed. Suggested role of: Automobile sheltering	Consecutive case series
Shibata M, 2014	2011 Japan earthquake and tsunami	269	24% Calf DVT	NR	Questionnaire Physical exam Calf ultrasonography	1 m	Lower limb trauma Reduced frequency of urination Sleeping in a vehicle	Consecutive case series
Sakuma M, 2006	2004 Mid Niigata earthquake	117	NR	10 cases	Questionnaire CT Population divided in high (>5%) and low evacuee area	1 m	High evacuee Female Suggested role of automobile sheltering	Retrospective analysis
Shibata M, 2017	2011 Japan earthquake and tsunami	3316	11.85% distal DVT	NR	US	26 m	-Older -Symptomatic -Female -VTE history -Attempting to perform daily exercise	Consecutive case series
Tauqir SF, 2007	Pakistan 2005 earthquake	194	2%	1 case	US	2 m	NR	Cross-sectional retrospective
Guner SI, 2014	2011 Van earthquake	46 affected by crush syndrome	NR	1 case	US, CT	NR	NR	Descriptive analysis
Sueta D, 2017	2016 Kumamoto earthquake	1	NR	1 case	CT	NR	-Vehicle sheltering -Oral contraception	Clinical case
Aoki T, 2013	2011 Japan earthquake and tsunami	NR	52 not specified pulmonary thromboembolism cases		NR	1 y	NR	Retrospective analysis
Ueda S, 2012	2011 Japan earthquake and tsunami	8630	2.2%	NR	US	3 m	-Flooded shelters	Consecutive case series
Rathore MF, 2008	Pakistan 2005 earthquake	187 survivors with acute spinal injury	4.8%	NR	US	2.5 m	-No influence of age, gender (P=0.4), spinal injury grade, fracture	Prospective observational
Matsukawa M, 2018	Kumamoto 2016 earthquake	76	32	54	US, CT	4 m	NR -DOACS found to be safe and effective in reducing recurrence, without increasing bleeding	Prospective observational
Modena MG, 2017	2012 Modena earthquake	1401	DVT-PE reported together: 64 vs 9 of the year before		US, CT	1 y	Female	Retrospective analysis
Groves CC, 2017	2015 Nepal earthquake	117 spinal cord injuries	6%	NR	US	14 m	NR	Descriptive
Terakami T, 2009	2007 Ishikawa earthquake	198	10.6%	NR	Questionnaires US D-dimer	NR	NR	Descriptive
Ueda S, 2014	2011 Japan earthquake and tsunami	701	190	NR	US	9 m	-Flooded shelters -Temporary emergency houses	Consecutive case series

DVT: deep venous thrombosis; PE: pulmonary embolism; F-UP: follow-up; NR: not reported; m: months; CT: computed tomography; US: ultrasound; y: year; DOACS: direct oral anticoagulants; VTE: venous thromboembolism

The total study population in this review included 16,916 subjects who were exposed to the earthquake. In three papers, information on the number of subjects was not reported. Mean follow-up after the disaster was 6 months, ranging from 1 to 26 months. No further details were provided regarding gender, age, and comorbidities distribution. No randomized comparative trials were found on the topic. All the identified investigations were consecutive case series or cross-sectional retrospective analysis.^11–27)^

An assessment of homogeneity at baseline of the herein reported scientific works was not feasible. Following the 2020 Cochrane Handbook for Systematic Reviews of Interventions publication on how to report risk of bias in scientific literature review,^28)^ an evaluation of the bias risk assessment for the reviewed papers was performed: the reviewed publications showed lacking random sequence generation and allocation concealment, partly due to the unpredictable nature of the disaster and partly because of lacking study design (e.g., possible randomization in use of graduated elastic stockings for thrombosis prevention following an earthquake).

Blinding of participants, personnel was missing as well. Outcome assessment was deemed to be extremely incomplete, as exemplified by the lack of screening for distal deep venous thrombosis rather than full leg assessment. The vast presence of these biases made the creation of a detailed risk bias table futile, considering all the investigations would have resulted in high risk of bias.

### Main findings on earthquake impact on venous disease

In 2006, Sakuma et al. have reported an increased incidence of pulmonary embolism following the 2004 Mid Niigata Prefecture earthquake in Japan. The investigation focused on the high pulmonary embolism rate in evacuees. Moreover, female gender and the use of the automobile as night shelter were found to be potentially associated with increased venous thromboembolic risk.^15)^

The role of automobile sheltering in thromboembolism was highlighted by the Inoue in 2006, following their analysis of the 2004 Chuetsu District earthquake.^13)^

In 2007, the analysis of Tauqir et al. on the 2005 earthquake in Pakistan has pointed out an increased percentage of venous thrombosis in the population hit by the disaster, but no risk factor analysis was performed.^23)^ Following the same event, Rathore et al. focused on 187 patients with spinal cord injuries due to disaster-related direct trauma. No influence on the thrombotic risk was reported for age, gender, spinal injury grade, or eventual fractures.^19)^ Examining the patients affected by post-traumatic spinal cord injuries, Groves et al. have confirmed the increased venous thrombosis incidence after the 2015 earthquake in Nepal.^26)^ In 2008, Watanabe et al., who conducted a study on the 2004 Niigata earthquake, confirmed the possible role of automobile sheltering in the increased incidence of pulmonary embolism.^12)^ In 2009, Terakami et al. reported a significantly increased deep venous thrombosis incidence rate (10.6% of the affected population) following the 2007 Ishikawa earthquake.^27)^ In 2012, Ueda et al. pointed out the role of flooded shelters on the increasing risk of venous thrombosis after the 2011 Japan earthquake and tsunami.^18)^ The Shibata analysis on the same event highlighted lower limb trauma, reduced frequency of urination, and sleeping in an automobile as conditions associated with a significant increase in calf deep venous thrombosis.^14)^ In 2014, another study from Ueda confirmed the role of flooded shelters and temporary emergency housing in the increased venous thrombosis rate after the 2011 Japan earthquake.^21)^ A subsequent publication of Shibata analysis of the same 2011 Japan earthquake confirmed advanced age and the female gender as potential risk factors for venous thrombosis following the geophysical disaster. In the same publication, potential risk factors were also identified in the symptomatic cases as well as those with a history of venous thromboembolism.^16)^ Pulmonary embolism risks were analyzed by Sueta et al. on the Kumamoto 2016 earthquake, wherein it was confirmed that automobile sheltering and oral contraceptives are potential risk factors.^17)^

Matsukawa et al. have also examined the same catastrophic event, but focusing on the use of direct oral anticoagulants in post-disaster thromboprophylaxis, highlighting their safety and potentials in reducing thrombotic recurrence, without increase in bleeding.^25)^ In 2019, Sato et al. assessed 1663 subjects exposed to the Kumamoto 2016 earthquake and reported a deep venous thrombosis incidence of 10.3%; potential risk factors identified were as follows: age >70, use of sleep medication, edema, and varicose veins.^11)^

### Main findings on how burns and intoxications impact venous disease

**Table 2** presents the results of the search on the topic of how burns and intoxication impact venous thromboembolism risk. Potential risk factors for venous thrombosis following burns were identified as follows: wound infections, prolonged hospital stay, obesity, total body surface area affected by burn, prolonged immobility, and D-dimer elevation.^29–31)^ Harrington et al. have looked into the risk of pulmonary embolism after burns, wherein advanced age and total body surface area were identified as two potential factors.^32)^ Organophosphate or carbon monoxide intoxication was categorized in the disaster category as “man-made” event, following, for example, an industrial accident. A longitudinal cohort study demonstrated the increased risk of both deep venous thrombosis and pulmonary embolism associated with organophosphate intoxication.^33)^ A retrospective case-control study demonstrated carbon monoxide intoxication was associated with an increased risk only of venous thrombosis.^34)^

**Table 2:** Table 2 Selected publications dealing with burns and intoxication impact on venous disease

First author, year	Disaster	Population	DVT	PE	Assessment tools	F-up	Identified risk factors	Study design
Wahl WL, 2001	Burns	327	2.4%	NR	US	NR	-Infections -Hospital length of stay	Review
Harrington D, 2001	Burns	1300	2.9%	2.9%	US, CT	5.5 y	-Age -TBSA	Retrospective analysis
Ahuja RB, 2016	Burns	50	8%	NR	US	Not specified	-BMI -TBSA -Prolonged immobility -Longer duration of stay -D-dimer (day 5)	Randomized controlled trial
Lim YP, 2015	Organophosphate intoxication	9223	Adjusted hazard ratio=1.55	Adjusted hazard ratio=1.44	US	11 y	OP intoxication	Longitudinal cohort study
Chung W, 2015	Carbon monoxide poisoning	8316	3.85-fold higher risk of DVT	Not significantly associated with risk of PE	US, CT	11 y	Carbon monoxide intoxication	Retrospective case-control
Shen C, 2017	Alcohol intoxication	61,229	Risk of DVT=3.40 higher	Risk of DVT=3.53 higher	US, CT	10 y	Alcohol intoxication	Retrospective case-control

DVT: deep venous thrombosis; PE: pulmonary embolism; F-UP: follow-up; NR: not reported; CT: computed tomography; US: ultrasound; y: year; BMI: body mass index; TBSA: total body surface area; OP: organophosphate intoxication

On the other hand, alcoholism should also be considered as a form of intoxication given its high prevalence in the society resembling a form of disaster.^35)^ In a large population study, Shen et al. demonstrated an increased risk of both deep venous thrombosis and pulmonary embolism associated with alcoholism.^36)^

## Discussion

A 2019 review has reported the association of earthquakes with a number of cardiovascular events: mainly sudden cardiac death, myocardial infarction, cardiomyopathy, heart failure, stroke, arrhythmias, hypertension, and pulmonary embolism. The increased incidence was evident in post-disaster time, ranging from few hours up to several months.^37)^

Disasters’ effects on the cardiovascular system were reported even 10 years after Hurricane Katrina.^38)^

Subjects hit by earthquakes and natural disasters often have be evacuated to emergency shelters, which, in turn, could lead to hypomobility and poor hygiene. In particular, crowded and/or flooded shelters can increase the risk of contagious diseases, as reported by Kawano research group following the Great Eastern Japan earthquake.^39)^ In this sheltering scenario, acute respiratory infections and acute gastroenteritis were the most frequently reported conditions.^40)^

In these times of pandemic brought about by COVID-19, proper knowledge on the literature related to the topic and “preparedness” for facing the eventual disaster emergency is of paramount importance.

Indeed, sheltering in these times could represent an extremely challenging situation for an already strained public health system.^41)^ This difficulty becomes even more evident as COVID-19 has been clearly associated with an increased risk of venous thrombosis per se,^42)^ with 4.5% bilateral deep venous thrombosis reported even in patients hospitalized in non-intensive care units.^43)^ Among the investigated disasters, earthquakes was determined to be the most common topic, with its significant increase in terms of risk for venous thrombosis. Experiencing sheltering after an earthquake in this pandemic would definitely stretch preparedness at multiple levels. An example of the need of having sonographers ready to detect the eventual thrombosis was nicely reported already by Shimura.^44)^ A multispecialty approach to the disaster management was nicely outlined by Hata, focusing on the central role of the general practitioners as health professionals, as they are aware of patients’ risk factors, particularly in case of pro-thrombotic post-disaster scenarios.^45)^ From the herein review, the same pro-thrombotic nature of the post-earthquake condition became as evident as the need of properly organized shelters allowing proper movement, distancing, and hygiene to their inhabitants. At the same time, the heterogeneity encountered in the currently available data collections on the topic rendered a meta-analysis impossible to be performed. The authors involved in all the assessed papers have to be congratulated for both their scientific effort and the will of collecting data in such a challenging context for the benefit of all, especially, the ones who will have to face upcoming disasters. The herein presented data suggest the importance of clear protocols of data collection for venous thromboembolism incidence assessment and related risk factor identification. An example of the unmet evidence-based need in this current literature is the lack of ultrasound screening performed along the limb rather than just at the calf region. While the below knee scanning demonstrated to be of great clinical use, particularly considering the difficulty of performing a whole leg scan in the real-world disaster environment,^16)^ future investigations aimed to collect data suitable for meta-analysis should follow the same scanning protocol. In the papers assessed in this review, no data were found on the prophylactic/therapeutic measures adopted for thrombosis control in the study population after a disaster, with the exception of the investigation of Matsukawa which examined the potential role of direct oral anticoagulants.^25)^

Particularly in pandemic time, the possibility of using drugs not requiring constant monitoring and blood sampling is deemed appealing. Nevertheless, as per this present review, proper studies specifying a homogeneous protocol including drugs and/or graduated compression stocking use for venous thrombosis prevention/treatment are missing in the disaster context. Family history of thrombosis was recognized as a potential risk factor for post-disaster thrombosis by Shibata et al.^16)^ This finding highlighted the importance of proper reporting of comorbidities in future investigations on the topic. Indeed, the currently available literature has failed to examine information as regards the personal thrombotic risk assessment of the subjects affected by thrombosis following a disaster.

A special focus should also be placed on the monitoring of patients affected by physical trauma following a disaster. According to Rathore paper, fractures and spinal injury grade were not associated with an increase in the thrombotic risk following the Pakistan 2005 earthquake.^19)^

Yet, Groves identified an increased thrombotic incidence in post-earthquake crash syndrome patients.^26)^ Special care should also be placed in the detection of fat embolism after a fracture: a condition underestimated following earthquakes as per a study conducted by Wang et al.^46)^ The potential benefits of proper rehabilitation following earthquake-induced physical trauma, including the venous thrombosis aspect, were described by Li et al.^47)^ Another aspect that should not be underestimated is the disaster impact on mental health.^48)^ One should always keep in mind that the most advanced stages of chronic venous disease are often characterized by a depressive symptomatology and related hypomobility, thus inducing a vicious circle that could lead to worsening lower limb function.^49)^ Chronic venous disease per se is a significant risk factor for venous thromboembolism.^50)^ As per the investigation published by Sato et al., varicose veins are considered a potential risk factor in the specific context of a disaster like the 2016 Kumamoto earthquake.^11)^ Future investigations should always include an assessment of the study population characteristics in terms of chronic venous disease comorbidities. Moreover, they should include the details of disease stage, in order to create more homogenous groups at the baseline of the investigation. The same attention should also be given to gender and age, as potential risk factors.^20)^

For example, in the context of the female gender, thrombotic risk can change significantly with menopause; thus, assessing gender without considering age may lead to potential bias.^51)^ The same need for homogenous data collection in order to generate strong evidence-based recommendations is important in the context of burn and intoxications as potential causes of thromboembolism. The amount of literature found on the topic in this review is extremely scarce, yet extremely interesting and useful for further research on the topic.

Indeed, as reported by Giarratano,^52)^ disaster research remained an open field, full of difficulties, but also of opportunities for valuable data collection in hopes to improve assistance to patients afflicted by both the disease and the disaster.

## Conclusion

Disasters are not rare events, and they require immediate action for containment due to devastating health threat. A literature body is identified by this review, pointing out the incidence of venous thromboembolism associated with earthquakes.

Burns and intoxications have only been partially investigated as potential risk factors for thromboembolism, but preliminary data clearly showed the need for further research on these topics considering their potential impact on venous health. Homogenous data collection protocols are needed in order to facilitate future meta-analysis on the topic of disaster and venous thromboembolism. The topic becomes of even greater importance considering the COVID-19 pandemic, its direct pro-thrombotic action and on top of a potentially catastrophic disaster, forcing those affected to aggregate in a poorly prepared shelter. The consequence is unimaginable.

## Notes

This is a review article based on the informative seminar of the 40th Annual Meeting of Japanese Society of Phlebology.
